# 90 YEARS OF PROGESTERONE: Molecular mechanisms of progesterone receptor action on the breast cancer genome

**DOI:** 10.1530/JME-19-0266

**Published:** 2020-06-02

**Authors:** Miguel Beato, Roni H G Wright, François Le Dily

**Affiliations:** 1 Centre de Regulació Genomica (CRG), Barcelona Institute of Science and Technology (BIST), Dr. Aiguader 88, Barcelona, Spain; 2 Universitat Pompeu Fabra (UPF), Barcelona, Spain

## Abstract

Gene regulation by steroid hormones has been at the forefront in elucidating the intricacies of transcriptional regulation in eukaryotes ever since the discovery by Karlson and Clever that the insect steroid hormone ecdysone induces chromatin puffs in giant chromosomes. After the successful cloning of the hormone receptors toward the end of the past century, detailed mechanistic insight emerged in some model systems, in particular the MMTV provirus. With the arrival of next generation DNA sequencing and the omics techniques, we have gained even further insight into the global cellular response to steroid hormones that in the past decades also extended to the function of the 3D genome topology. More recently, advances in high resolution microcopy, single cell genomics and the new vision of liquid-liquid phase transitions in the context of nuclear space bring us closer than ever to unravelling the logic of gene regulation and its complex integration of global cellular signaling networks. Using the function of progesterone and its cellular receptor in breast cancer cells, we will briefly summarize the history and describe the present extent of our knowledge on how regulatory proteins deal with the chromatin structure to gain access to DNA sequences and interpret the genomic instructions that enable cells to respond selectively to external signals by reshaping their gene regulatory networks.

## Introduction

The steroid hormone progesterone was initially considered to be involved mainly in menstrual cycle, pregnancy, and mammary gland function. Meanwhile, it was also implicated in multiple other functions outside the sexual organs. In this review we will concentrate on the role of progestins (Pg) on the regulation of gene expression via their specific progesterone receptor (PR), focusing on breast cancer cells. All the steroid hormone receptors share a similar organization of domains – a central short DNA-binding domain composed of two zinc fingers coordinated each by four cysteines; a C-terminal domain that is responsible for hormone binding and interaction with co-regulators; and a N-terminal domain of variable length, which is mainly unstructured and includes multiple residues that can be post-translationally modified and fine tune the receptors’ functional activity (see (Gronemeyer 1992; Huang *et al.* 2010), for review on steroid hormone receptor structure). In the case of PR, alternative initiation of the same gene leads to the generation of two PR isoforms, the complete isoform named PRB and the PRA isoform, which lacks the first 164 amino acids (Kastner *et al.* 1990). The difference in the properties of these two isoforms has been a matter of debate as the results observed depended on the nature of the assays used, which mainly were *in vitro* assays or transient transfection experiments (Jacobsen and Horwitz 2012; Khan *et al.* 2012). However, the importance of the N-terminal region of PR for hormone action and some recent experiments point to a significant functional difference between the two isoforms that will be addressed in the last section of this review.

This review will mainly focus on the mechanisms that enable the PR to access DNA sequences on chromatin and to modulate the transcriptional rate of subsets of hormone responsive genes. Before going into the more recent results obtained with genome-wide approaches, we will summarize as a historical introduction the concepts elaborated at the turn of the last century based on the findings related to PR regulation of the MMTV expression.

## PR before omics: the MMTV promoter as a model system.

The role of progestins in breast cancer cells has been mostly neglected in favor of the more obvious effect of estrogens as drivers of cell proliferation (Carroll *et al.* 2017). This is particularly the case when we consider the most popular cell-culture model of luminal breast cancer, namely the MCF-7 cell line, which exhibits higher levels of estrogen receptor alpha (ERα) compared to PR. However, more recently, the interplay between estrogens and progestins has received considerable attention, predominantly based not only on results from cell-culture models but also on whole animal studies (Mohammed *et al.* 2015; Need *et al.* 2015; Singhal *et al.* 2016). Most studies on progestin action in cell culture have used the T47D cell line that, in contrast to MCF7 cells, expresses much higher constitutive levels of PR compared to ERα.

**Figure 1:**
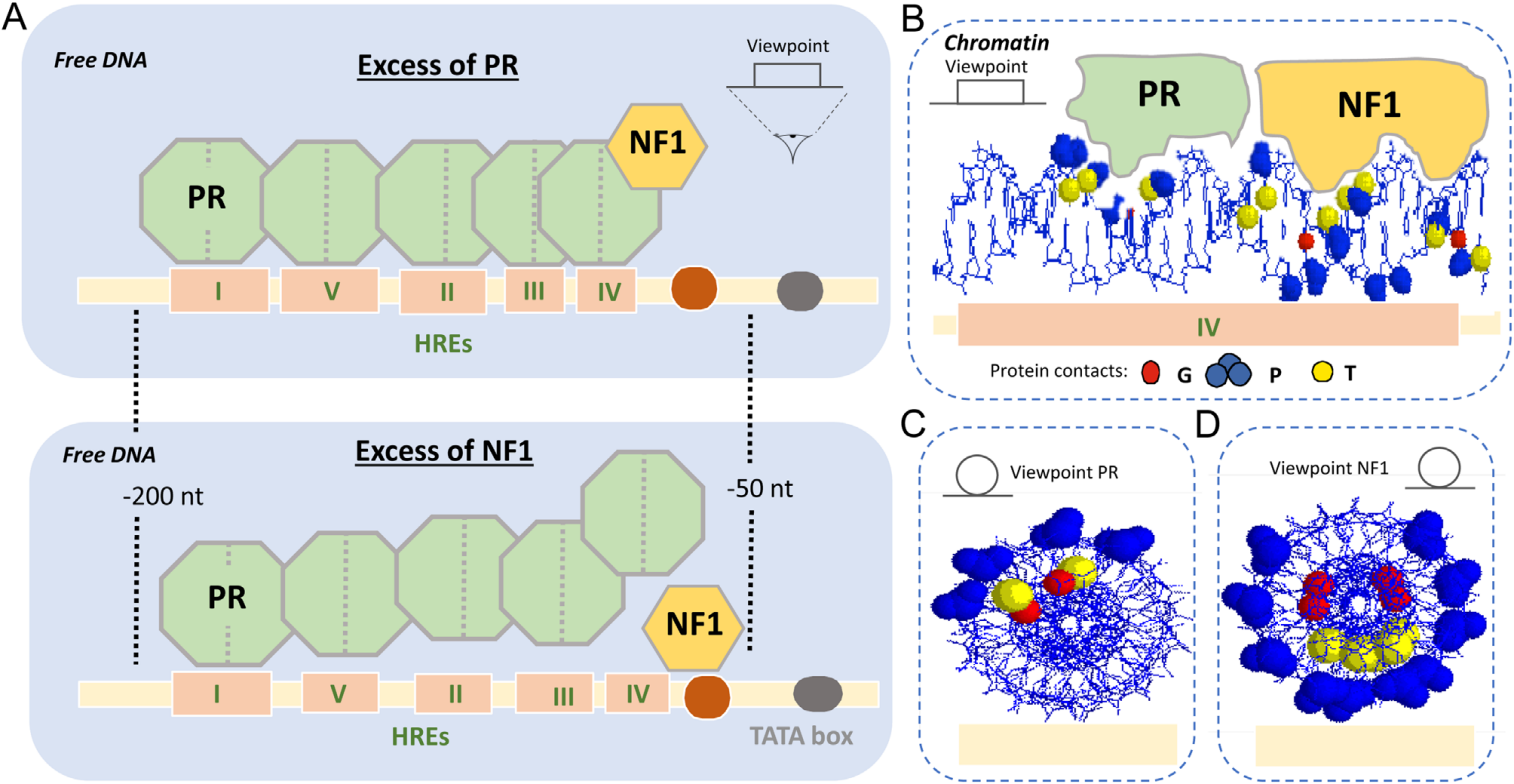
The hormone responsive region of the MMTV promoter. (A) Of the five hormone response elements (HREs) in the MMTV promoter, only HRE-I is a perfect palindromic sequence. The other four HREs are half palindromic, although in *in vitro* DNA binding experiments homodimers of the PR bind cooperatively to each HREs. The nucleotides upstream of the transcription start sites and the position of the TATA box are indicated (Scheidereit *et al.* 1983; Chávez & Beato 1997). PR and NF1 compete for binding to the MMTV promoter DNA *in vitro*. The presence of an excess of PR or preincubation with PR precludes NF1 binding (*top*). In contrast, the presence of an excess of NF1 or preincubation with NF1 diminishes PR binding (*bottom*). This is due to the proximity of the HRE-IV and the NF1 binding sequence and to the cooperative binding of PR to all HREs (Bruggemeier *et al.* 1990). PR can bind its target sequence in nucleosomes while NF1 cannot. Panel (B) shows the contacts of PR with the HRE IV and of *NF1 with* its target sequence in a side view and panels (C) and (D) in a view along the DNA helix axis taking PR and NF1 as view points, respectively. Contacts with guanines (G), phosphates (P) and thymines (T) are highlighted. The non-accessible contacts are those facing the core histone (Scheidereit & Beato 1984)

Prior to the establishment of whole genome approaches, most of the studies on PR action were based on the MMTV provirus model, which was initially used to study regulation by the glucocorticoid receptor (GR) (Ringold *et al.* 1975). Later, MMTV expression was shown to be regulated by Pg via the PR (Ahe *et al.* 1985). Both GR and PR were shown to bind cooperatively to DNA sequences of the MMTV LTR, upstream of the TATA box of the main MMTV promoter (Payvar *et al.* 1983; Scheidereit *et al.* 1983) (Fig. 1A). Similar sequences were found in many other Pg-regulated genes, although at different locations relative to the gene promoter. Comparison of these sequences led to the identification of the 15-mer palindromic hormone response element (HRE) with the consensus GGTACAnnnTGTTCT, which is recognized by a homodimer of PR in a head-to-head orientation (Beato 1989). The same 15-mer was also bound by GR, androgen receptor (AR) and the mineralocorticoid receptor. In the region bound by PR on the long terminal repeat (LTR) of the MMTV provirus, one canonical palindromic HRE and four incomplete HREs containing only one half of the palindromic sequence were identified. Downstream of the cluster of receptor-binding sequences is a palindromic binding site for the transcription factor nuclear factor 1 (NF1), which is important for efficient hormonal regulation. Intriguingly, PR and NF1 synergize in cell assays (Truss *et al.* 1995) but compete for binding to free DNA (Bruggemeier *et al.* 1990), suggesting that free DNA is not a sufficient template to completely explain the mechanism of hormonal gene regulation. This observation on free DNA led us to investigate the manner in which the MMTV promoter is organized in the context of chromatin.

When assembled in chromatin *in vitro* and in cells, the MMTV promoter positions precisely one nucleosome that includes the five HREs and the NF1 binding site (Richard-Foy and Hager 1987; Piña *et al.* 1990). The orientation of the DNA major groove in the surface of the histone octamer enables binding of PR to the palindromic HRE-I and to the half palindromic HRE-IV. This is possible because PR only contacts a narrow sector of the DNA double helix (Fig. 1B and C). In contrast, NF1 cannot bind its target sequence when assembled in nucleosomes as it contacts the whole circumference of the DNA helix (Fig. 1B and D) (Scheidereit and Beato 1984). However, in cells carrying an integrated copy of the MMTV promoter (Truss *et al.* 1995) or when assembled on the surface of MMTV minichromosomes (Di Croce *et al.* 1999), PR and NF1 synergize in an ATP-dependent process upon exposure to hormone. Thus, efficient MMTV induction depends on the proper nucleosome organization of its promoter, as demonstrated by the lack of synergism in histone-depleted yeast (Chávez and Beato 1997). This seems paradoxical, as the DNA binder with the weaker DNA affinity (PR) recognizes its target sequence on nucleosomes and upon ATP-dependent remodeling enables the occupancy of the stronger DNA binding factor (NF1) (Bruggemeier *et al.* 1990). However, something similar also occurs with the classical pioneer factors, such as FOXA1, which exhibit an even weaker affinity for DNA, compared to PR, but are capable of enabling binding of other transcription factors even in the absence of ATP (Zaret and Carroll 2011; Zaret *et al.* 2016).

With MMTV minichromosomes assembled in *Drosophila* embryo extracts, the synergism between PR and NF1 is improved in the presence of histone H1, which represses transcription by each of the factors added separately (Koop *et al.* 2003). The reason being that the presence of histone H1 increases the proportion of chromatin templates that adopt the correct nucleosome organization. During the transcription process, H1 becomes phosphorylated in a PR-dependent manner and is partially displaced from the chromatin template by the NURF complex (Koop *et al.* 2003). Thus, histone H1 fulfills a complex and dynamic role in the regulation of the MMTV promoter, keeping the promoter silent in the absence of hormone and enabling efficient induction in response to hormone. Later we found that MSK1 phosphorylation at S10 of histone H3 and the displacement of H2A/H2B dimers from the MMTV promoter nucleosome by the SWI/SNF complex are part of the chromatin remodeling required for MMTV activation (Vicent *et al.* 2004; Vicent *et al.* 2006). Thus, PR-induced transcription of the MMTV promoter is initiated on the surface of H3/H4 tetramer that is more sensitive to DNAse I at its dyad (Vicent *et al.* 2010).

## The PR cistrome in breast cancer cells

**Figure 2:**
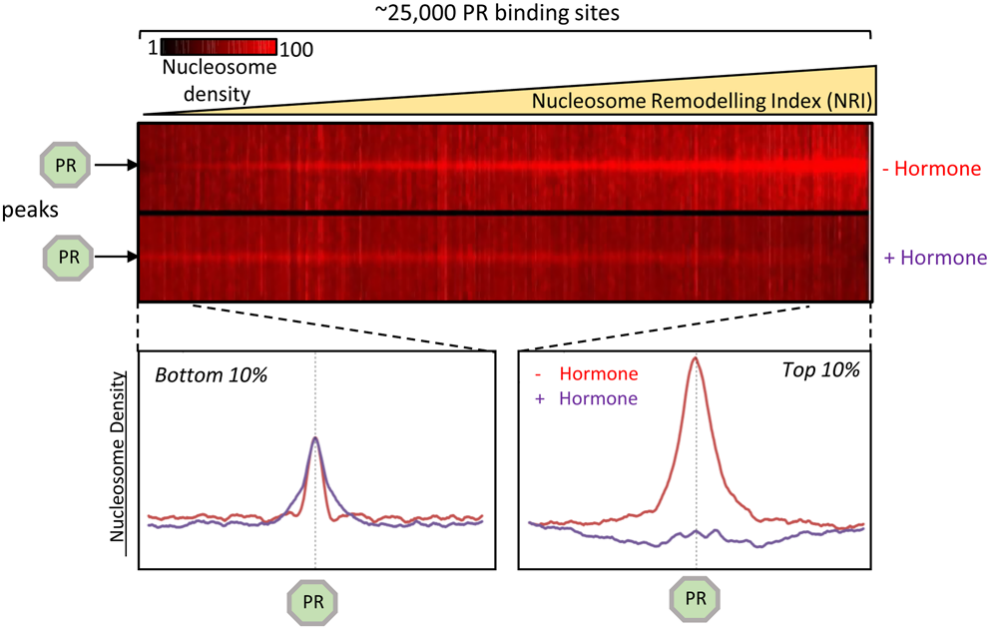
Classification of the genome PR**-binding sites according to their Nucleosome Remodeling Index (NRI). *Top panels*: Around 25,000 PR-binding sites detected upon 60 min of hormone exposure were classified according to their Nucleosome Remodeling Index (NRI) and are shown as a heat map of nucleosome reads before and after hormone exposure ordered from the highest NRI to th*e lowest* NRI. *Bottom panels*: *Right*: Nucleosome occupancy plot around the 2500 PR-binding sites with the highest NRI, before hormone exposure (red) and after 60 min of hormone exposure (lilac). *Left*: A similar nucleosome occupancy plot with the 2500 PR-binding sites with the lowest NRI (Ballare *et al.* 2013).

The arrival of whole genome approaches using next generation DNA sequencing made it possible for the first time to explore the global landscape of PR occupancy over the entire genome and to correlate this with the changes in the transcriptome upon hormone exposure. In T47D cells only a few hundred weak PR-binding sites were detected by chromatin immunoprecipitation and sequencing (ChIP-seq) with a PR-specific antibody prior to hormone exposure. In contrast, upon 30–60 min of exposure to 10 nM promegestone (R5020, abbreviated as Pg), we identified around 25,000 PR-binding sites and observed significant changes in expression of around 2000 genes using RNA-seq (Ballare *et al.* 2013). Sequence analysis revealed that most of these PR binding sites do not exhibit a complete palindromic HRE, but just one or several half HREs. As measured by MNase-seq, the large majority of PR binding sites were located over regions of chromatin enriched in nucleosomes prior to hormone exposure (Ballare *et al.* 2013) (Fig. 2, left panel, − hormone). However, after 30–60 min of hormone exposure, we found a significant decrease in the number of MNase reads over the PR binding sites, suggesting that the nucleosomes have been remodeled and become sensitive to MNase digestion (Ballare *et al.* 2013) (Fig. 2, left panel, + hormone). It is worth noting that other ligands binding to PR induced different binding dynamics and gene regulation. While Pg-occupied PR binds rapidly and exchanges rapidly as well, PR occupied by the partial agonist RU486 binds and exchanges more slowly, while PR occupied by the full antagonist ZK98299 does not bind to the MMTV promoter (Rayasam *et al.* 2005).

The extent of nucleosome remodeling was estimated by the ratio of MNase reads before and after hormone exposure, which we named Nucleosome Remodeling Index (NRI). Classifying the PR-binding sites according to their NRI (Fig. 2, left panel) showed that sites with the highest NRI corresponded to the strongest PR peaks that were associated with hormone responsive genes and were heavily remodeled upon hormone exposure (Fig. 2, right top panel). On the contrary, PR binding sites with the lowest NRI showed weaker peaks not associated with hormone responsive genes that were not remodeled upon hormone exposure (Fig. 2, right lower panel) (Ballare *et al.* 2013). Therefore, the genome-wide analysis of PR binding confirmed the original observation made with the MMTV model system years earlier, supporting that optimal PR binding, as observed in around 2500 high NRI sites, requires the PREs to be organized in an accessible way on nucleosomes that are remodeled upon hormone exposure. These findings are in conflict with a very general assumption that for transcription factor access to their DNA target sequences requires the previous displacement of nucleosomes. This idea does hold true for factors like NF1 that interact with DNA embracing the double helix, but does not apply to PR that only contacts a narrow sector of the DNA helix, as the distance between the two halves of the palindrome is 10 bp, both halves are similarly exposed on the surface of a perfect HRE (Piña *et al.* 1990) (Fig. 1B).

In the case of estrogens and glucocorticoids, it was reported that binding of their ligand-activated receptors to DNA occurs mainly at chromatin regions that are already accessible prior to hormone exposure, judged by the hypersensitivity of these regions to nucleases (John *et al.* 2011; Zaret and Carroll 2011). This is mainly attributed to the action of the pioneer factor FOXA1, which is postulated to prepare the chromatin for subsequent hormone receptor binding (Zaret and Carroll 2011). GATA3 could also act upstream of FOXA1 to enable ERα binding to its target sequences (Theodorou *et al.* 2013). Pioneer factors may remain attached to chromatin even during mitosis, enabling transcriptional memory (Palozola *et al.* 2019). In line with this, we have also uncovered that a fraction of the PR binding sites are already highly occupied by FOXA1 prior to hormone exposure and that the amount of FOXA1 does not increase after adding hormone, while a larger proportion of sites is marked by FOXA1 and ligand-activated PR favors further recruitment of FOXA1 (Nacht *et al.* 2019). However, we interpret the requirement of FOXA1 for PR binding as a consequence of FOXA1-mediated displacement of histone H1 (Iwafuchi-Doi *et al.* 2016) rather than to the removal of nucleosome core particles. In addition, we cannot exclude that these sites may reflect loops formed by distant interactions (Glont *et al.* 2019). Interestingly, we found a subset of PR binding sites that are assisted by C/EBPα binding prior to hormone exposure (Nacht *et al.* 2019). These regions exhibit epigenetic marks of active enhancers and C/EBPα favors contact of these enhancers with their target promoters by facilitating binding of RAD21, YY1 and the mediator complex (Nacht *et al.* 2019). In these sites, C/EBPα acts as a modulator of progestin action, enabling only a single round of cell division and slowing down the growth of xenografted cells (Nacht *et al.* 2019). A similar facilitating role of C/EBPα had been described for GR recruitment to the genome (Grontved *et al.* 2013). In endometrial cancer cells, binding of PR occurs in regions that are pre-marked by PAX2 binding (La Greca *et al.* 2019). These findings support the notion that gene regulation in differentiated cells depends on the combined action of various cell and sequence-specific transcription factors acting sequentially or simultaneously to fine-tune the gene expression program required.

## Chromatin remodeling and gene regulation in response to hormone

**Figure 3:**
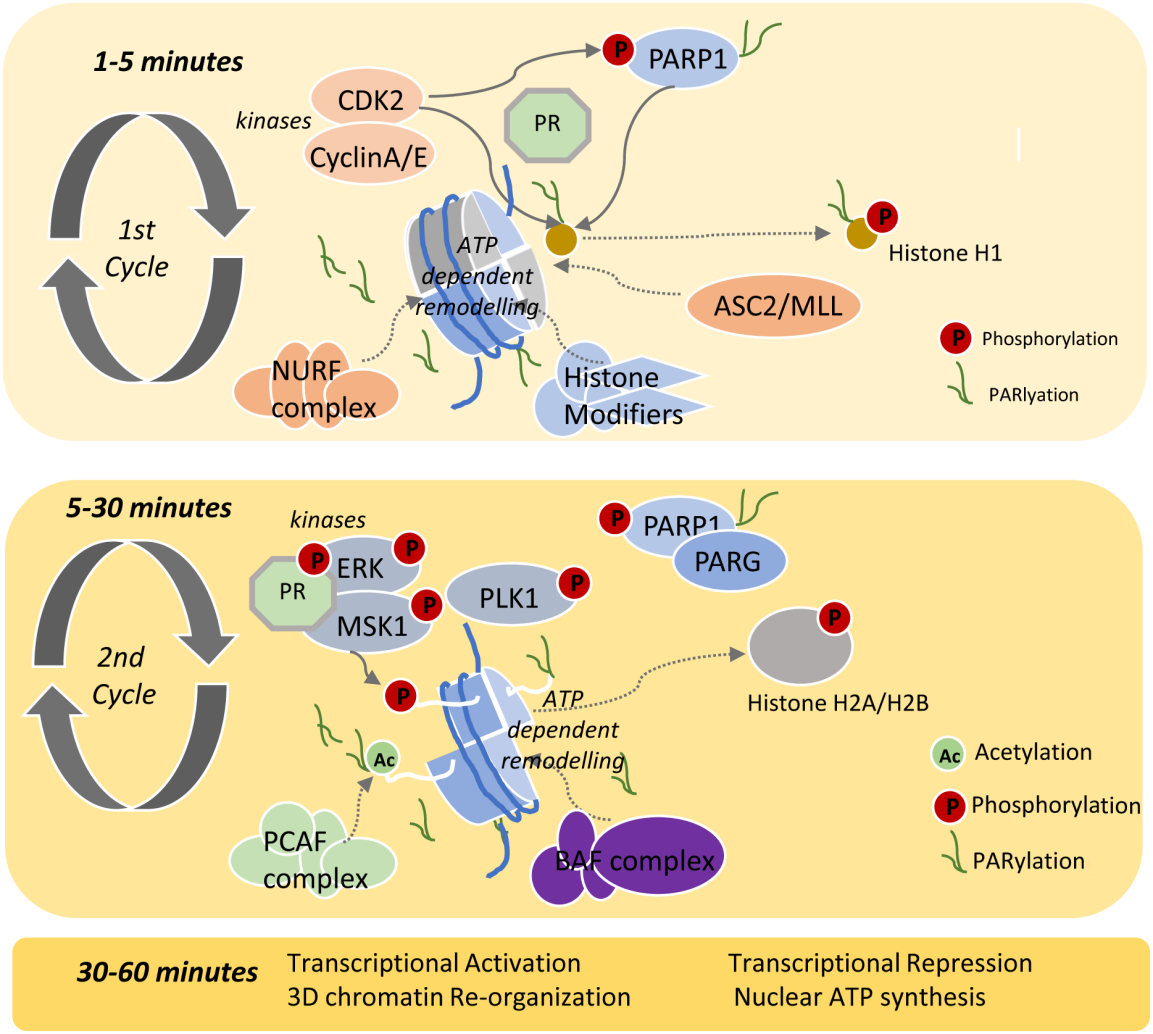
The two consecutive cycles of chromatin remodeling in response to hormone. The first cycle (*Top panel*) is rapid and leads to histone H1 phosphorylation and PARylation, followed by its displacement from chromatin. *Middle panel*: The second cycle is slower and leads to the displacement of histone H2A/H2B dimers. The enzymes involved in nucleosome remodeling and chromatin modifications are indicated. *Bottom panel*: functional consequences of chromatin remodeling (CDK2, Cyclin-Dependent Kinase 2; PARP1, Poly (ADP-ribose) polymerase 1; NURF, Nucleosome Remodeling Factor; ERK, Extracellular signal-Regulated Kinases; MSK1, Mitogen and Stress activated protein Kinase-1; PLK1, Polo-Like Kinase 1; PARG, Poly ADP-Ribose Glycohydrolase; PCAF, P300/CBP-Associated Factor; BAF, BRG1- or BRM-Associated Factors).

How does hormone binding to PR promote nucleosome remodeling? In that respect, it is important to note that hormone-induced chromatin remodeling and gene expression is also dependent on the activation of PR attached to the cell membrane. This small fraction of the PR is indeed attached to the cell membrane via cysteine palmitoylation (Pedram *et al.* 2007) and forms a complex with ERα and Src (Migliaccio *et al.* 1998). Upon binding of progestin, the membrane-attached PR activates Src, either directly (Boonyaratanakornkit *et al.* 2001) or via ERα (Ballare *et al.* 2003), leading to the activation of the SRC/ERK/MSK1 kinase pathway. Activated ERK1–2 phosphorylates PR that translocates to the cell nucleus in the form of a ternary complex PR/extracellular signal-regulated kinases 1 and 2 (ERK1–2)/Mitogen- and stress-activated protein kinase-1 (MSK1), which once located at Pg target genes phosphorylates histone H3 at S10 (Vicent *et al.* 2006). This step is crucial for the regulation of gene expression. Later we discovered that many more enzymes and steps are involved in hormone-induced nucleosome remodeling and gene activation, a process that can be divided into two consecutive cycles (Vicent *et al.* 2009; Vicent *et al.* 2011; Wright *et al.* 2012). The first cycle takes place within the first 1–5 min after hormone exposure, involves CDK2/CyclinA-mediated phosphorylation and activation of poly (ADP-ribose) polymerase 1 (PARP1), the MLL complex and the NURF complex. Ultimately these enzymes lead to the phosphorylation and PARylation of histone H1 (H1), resulting in its displacement from chromatin (Fig. 3, left panel). The second cycle takes place between 5 and 30 min after hormone exposure and requires MSK1, the histone acetyl transferase PCAF and the BAF ATP-dependent chromatin remodeling complex, leading to additional nucleosome remodeling and the displacement of histone H2A/H2B dimers (Fig. 3, right panel) (Vicent *et al.* 2006; Yang *et al.* 2007). Therefore, the actual activation of transcription takes place on a histone H3/H4 tetramer (Vicent *et al.* 2010), which can be cleaved by MNase, explaining the decrease in the density of nucleosome reads upon hormone exposure (Fig. 2). Later, we discovered that hormone-activated chromatin remodeling also requires nuclear synthesis of ATP from ADP-Ribose and PPi by NUDIX5 (Wright *et al.* 2016) (see subsequent section).

Most of what has been said previously was discovered by studying hormonal gene activation, but the addition of hormone to breast cancer cells also represses a substantial number of genes (Ballare *et al.* 2013). Curiously, around 20% of the genes that will be activated by progestins are initially silenced by a repressive complex recruited by unliganded PR and contain heterochromatin protein 1-γ (HP1γ), lysine-specific histone demethylase 1 (LSD1), histone deacetylase 1 and 2 (HDAC1/2), REST corepressor 1 (CoRest), lysine demethylase 5B (KDM5B) and the steroid receptor RNA activator (SRA). Upon hormone exposure and as a result of H3S10 phosphorylation by MSK1, the HP1γ -LSD1 repressive complex is displaced, enabling the recruitment of additional co-regulators needed for full de-repression (Vicent *et al.* 2013). In a subset of other genes, hormone exposure leads to the recruitment of HP1γ and Brahma-related gene-1 (BRG1) resulting in hormone-induced gene down-regulation (Vicent *et al.* 2013). In these genes, repression is mediated by BRG1-dependent deposition of linker histone H1.2 (Nacht *et al.* 2016). Likely, other mechanisms of hormone mediated gene repressions still remain to be discovered.

## Role of 3D genome structure

Beyond the first level of genome packaging in nucleosomes, higher levels of genome folding have entered the field of epigenetics due substantially to the development of chromosome conformation capture techniques (Dekker *et al.* 2002); particularly, the genome-wide detection of chromatin proximity by ligation using Hi-C (Lieberman-Aiden *et al.* 2009). Hi-C and the techniques derived thereof continue to provide information about the different levels of genome folding, from chromosome territories to chromosome compartments, topologically associated domains (TADs), sub-TADs and loops (Bonev and Cavalli 2016). Formation of TADs is accepted to be generated by the mechanism of loop extrusion mediated by Cohesins and controlled by architectural proteins, such as the CCCTC-binding factor (CTCF) (Fudenberg *et al.* 2016). The RNA-binding region of CTCF participates in self-association and loop formation (Hansen *et al.* 2019; Saldana-Meyer *et al.* 2019).

**Figure 4:**
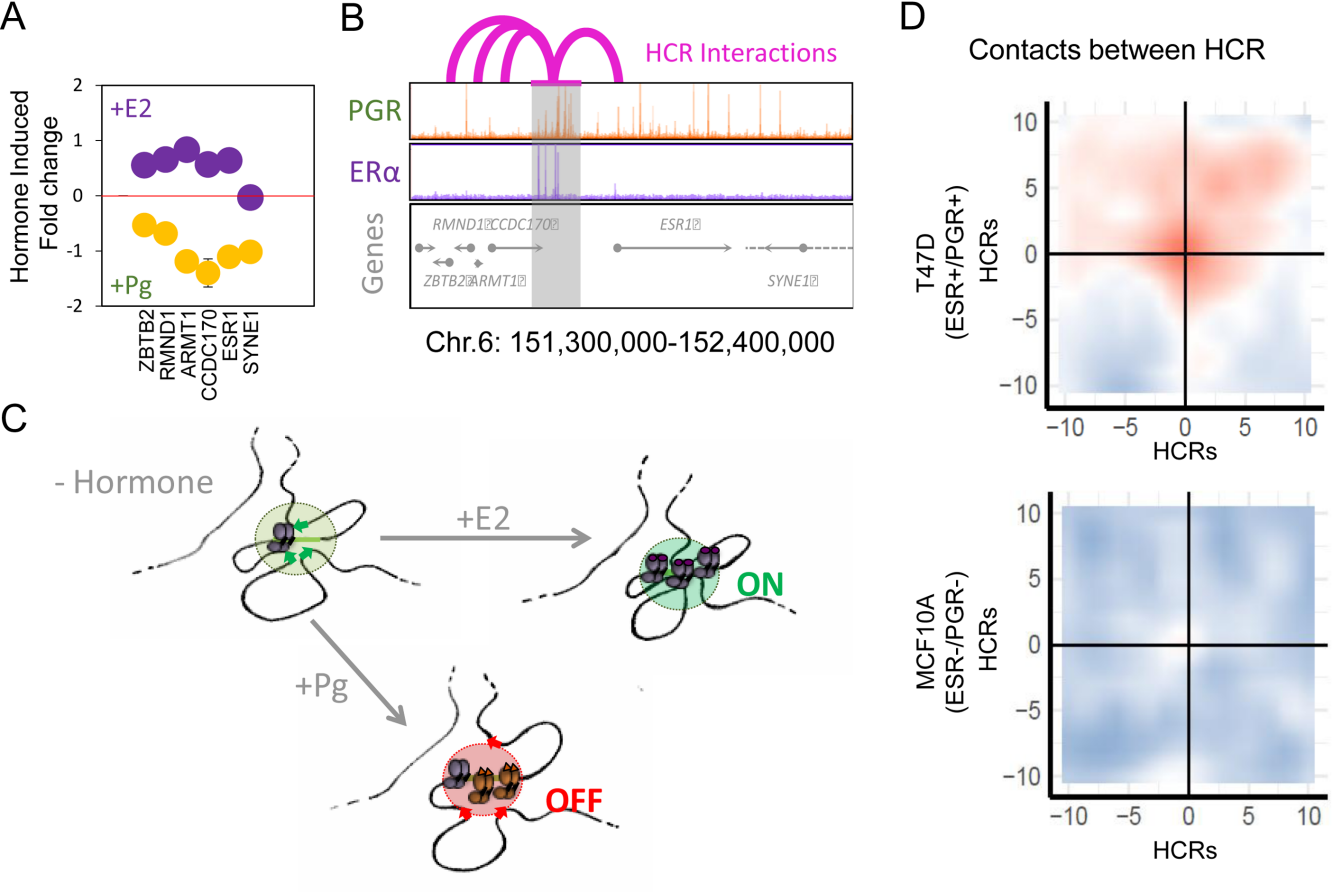
TADs are units of hormone response organized by hormone control regions (HCR) that interact with all genes in the TAD. (A) The TAD that includes the ESR1 gene contains five other genes, which are coordinately repressed by progestins (+Pg) but activated by estradiol (+E2). (B) The ESR1-containing-TAD is organized around an HCR (highlighted by the grey column) which corresponds to a cluster of binding sites of ERα and PR as detected by ChIP-Seq. The arcs on the top reflect the interactions established by this HCR with the promoters as detected by chromosome conformation capture methods. (C) The interactions HCR-promoters are enhanced upon binding of ERα after exposure to E2, whereas binding of PR after exposure to Pg weakens these interactions. (D) Analysis of long-range intra-chromosomal interactions between HCR containing TADs revealed that HCRs contact each other before hormone exposure in cells expressing ERα and PR (T47D), but not in similar cells negative for the receptors (MCF10A). The axis indicates the relative genomic distance expressed as function of the size of the HCR (X). The color scale from blue to red corresponds to the log_2_ ratio of observed frequencies of interaction between HCR over the contacts expected for random loci separated by the same genomic distances (Le Dily *et al.* 2019).

We have used Hi-C approaches to explore the role of genome topology in gene regulation by progestins. Curiously, we found that the division of the genome in TADs of around 1 Mb size is not affected by exposure of breast cancer cells to progestins, but that many of the hormone regulated genes are clustered in a subset of hormone responsive TADs. Moreover, all the coding and non-coding genes within a regulated TAD tend to respond in the same direction, suggesting that these TADs represent units of hormone response (Le Dily *et al.* 2014) (Fig. 4A). Hormone activated TADs expand in size and lose histones when exposed to hormone, while hormone repressed TADs become more compacted and enriched in histones (Le Dily *et al.* 2014). A more detailed analysis of a few interesting TADs revealed that all the gene promoters within a TAD interact with one single 20–90 kb long hormone control region (HCR) encompassing several PR- and ERα-binding sites. An example is the TAD containing the ERS1 gene encoding ERα, where the genes are activated by estrogens and repressed by progestins (Le Dily *et al.* 2019) (Fig. 4A and B). In response to estrogens (+E2), the HCR interactions with the promoters within the TAD are enhanced, whereas in response to progestins (+Pg), the HCR-promoters interactions are destabilized (Fig. 4C). We identified around 200 HCRs in T47D breast cancer cells and found that, prior to hormone exposure, the HCRs interact with each other at long distances at higher frequency than expected in cells expressing ERα and PR but not in receptor negative cells of the same epithelial luminal origin (Le Dily *et al.* 2019) (Fig. 4D). Thus, via the HCRs, the hormone receptors contribute to the higher order structure of the genome in a cell-type-specific manner and preform the conditions for an optimal hormone response. Similar structure has been proposed using Tri-C as regulatory hubs for mouse globin loci in primary mouse erythroid cells (Oudelaar *et al.* 2018).

One question that has not been sufficiently explored is the role of the underlying DNA sequence information on the higher order folding of chromatin. Ultimately, if chromatin folding is subjected to evolution, one would expect that the DNA sequence carries at least part of the information. However, except for the significance of repeated elements, very little has been established on the role of DNA sequence. In the filamentous fungus *Epichlöe Festucae*, blocks of repeated elements act as boundaries that organize the 3D genome structure and separate gene-rich genomic domains (Winter *et al.* 2018). In *Drosophila*, SINE elements have been shown to maintain and reshape 3D chromosome structure (Cournac *et al.* 2016). In the human genome, MIR and L2 transposable elements share long-range interactions, acting as enhancers that shape gene regulatory networks (Cao *et al.* 2019). Active Alu elements are very abundant in the human genome and have binding sequence for very relevant transcription factors (Bennett *et al.* 2008). In breast cancer cells, we have identified a class of Alu elements bound by the CHAP complex that, in response to serum starvation, reshape the 3D genome structure by binding of general transcription factor 3C (TFIIIC). TFIIIC acetylates H3 at K18 and forms loops that contact CTCF sites contained within cell cycle genes, maintaining them ready to respond to serum addition (Ferrari *et al.* 2019).

In addition to repeated elements, the biophysical properties of DNA not only influence the positioning of nucleosomes (Segal *et al.* 2006), but can also be used to identify special regulatory elements. Very recently, it has been shown that the increased DNA flexibility and accessibility of sequences with a high propeller twist are an optimal predictor of regulatory enhancer regions (Pataskar *et al.* 2019). Many years ago, Giorgio Bernardi proposed that the structure of chromosomes is determined by compositional DNA structure that determines what he now calls the genomic code. He first discovered that calf thymus DNA exhibits a reversible decrease in viscosity at sub-melting temperatures that affects −35% of the GC-poor DNA molecules (Freund and Bernardi 1963). Using chromatography of human DNA on hydroxyapatite columns and Cs_2_SO_4_/Ag^+^ density gradients, he established the concept of isochores, large DNA sequences, −0.9 Mb average size, that can be separated into five families of increasing GC content, decreasing size and increasing gene content, L1, L2, H1, H2, H3 (Macaya *et al.* 1976; Thiery *et al.* 1976). The isochore H3 with the highest GC content accounts for only −3% of the human genome and is very gene rich, constituting the ‘genome core’ (Bernardi *et al.* 1985). FISH experiments showed that the isochores with high-GC content are located in the center of the nucleus. Their size and location correspond to the TADs as defined in Hi-C experiments (Dixon *et al.* 2012; Nora *et al.* 2012). In contrast, the low-GC content isochores represent gene deserts and are associated to the nuclear lamina, corresponding to the lamina associated domains (LADs) (Guelen *et al.* 2008). The isochores behave as domains of DNA replication timing, with the short gene-rich isochores replicating early in S phase and the large gene-poor isochores replicating later (Costantini and Bernardi 2008). This bimodal distribution of the compositional genome structure is reminiscent of the two A and B chromosome compartments identified in Hi-C experiments (Lieberman-Aiden *et al.* 2009). Moreover, in the mouse genome interspersed GC-poor LINEs and GC-rich SINEs repeats constrain the base composition of isochores. These results suggest that there is a DNA-guided folding of chromatin, a kind of genome code, which could be the genetic basis for genome topology. For further reading on this interesting proposal, see Bernardi’s recent review (Bernardi 2019).

## Role of nuclear ATP synthesis

**Figure 5:**
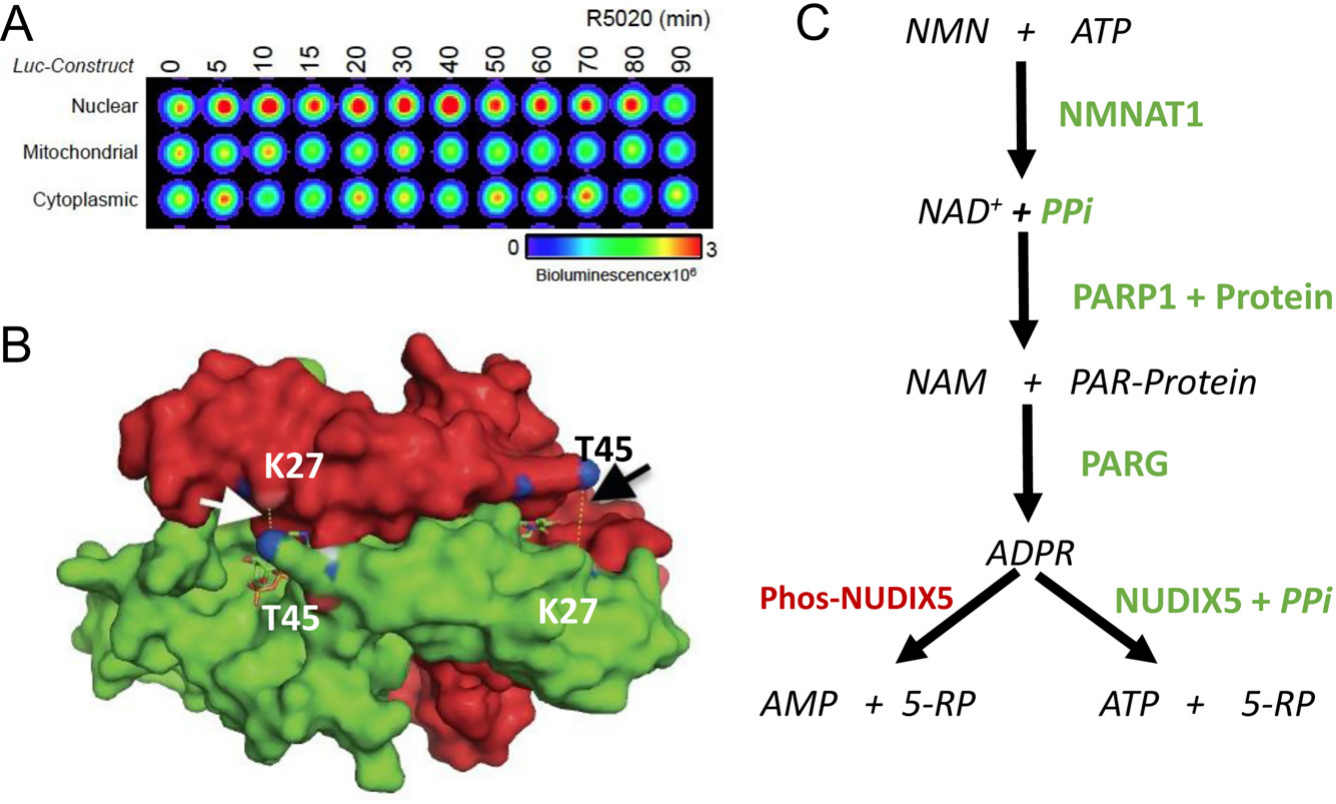
Nuclear, mitochondrial and cytoplasmic ATP measurements using bioluminescence reporters. (A) Images of ATP levels of T47D cells exposed to Pg for the indicated time periods, from cells expressing the luciferase reporters target to the cell nucleus, the mitochondria or the cytosol (based on data from Wright *et al.* 2016). (B) Hormone exposure activates NUDIX5 via rapid dephosphorylation of T45; surface of the crystal structure of the NUDIX5 T45D mutant homodimer complexed with ADPR and MG2+ (base on the native structure PDB: 2DSC). The two symmetrical contacts between T45D (blue) of one monomer and K27 of the other are highlighted. (Wright *et al.* 2016). (C) The nuclear ATP recovery nuclear pathway. The mitochondrial ATP used by NMNAT1 to make NAD^+^ is converted by PARP1 to protein-attached PAR, which is degraded by PARG into ADPR. ADPR can be hydrolyzed by phosphorylated NUDIX5 to AMP and ribose-5-phosphate, or by unphosphorylated NUDIX5 in the presence of PPi to ATP and ribose-5-phosphate (NMN, Nicotinamide Mononucleotide; NMNAT, Nicotinamide Nucleotide Adenylyltransferase 1; NAD, Nicotinamide Adenine Dinucleotide; PARP1, Poly (ADP-ribose) polymerase 1; NAM, Nicotinamide; PAR, Poly ADP-ribose; PARG, Poly ADP-Ribose Glycohydrolase; NUDIX5, NUcleoside Diphosphate Linked Moiety X).

As mentioned previously, one of the key steps involved in progestin signaling is the rapid activation of PARP1. PARP1 is activated in response to hormone via CDK2 mediated phosphorylation within its NAD^+^ binding active site. Activation in this manner increases subsequent nuclear poly(ADP-ribose) (PAR) (Wright *et al.* 2012). Blocking this step via inhibition of PARP1 prevents the regulation of 80% of the hormone-controlled genes and inhibits chromatin remodeling and histone H1 displacement. Initially, we assumed that the increase in PAR synthesis and the PARylation of histones was only needed for chromatin decompaction that would facilitate access for transcription factors, other histone modifying enzymes and cofactors to ensure correct transcriptional regulation (Wright *et al.* 2012). Unexpectedly, however, we found that the hydrolysis of PAR to its ADPR subunits by poly ADP-ribose glycohydrolase (PARG) was also required for hormonal gene regulation, a finding that led us to think about other possible functions of ADPR in hormone regulation (Wright *et al.* 2016), a concept that was initially formulated in the context of DNA repair (Maruta *et al.* 1997; Oei and Ziegler 2000; Maruta *et al.* 2007). Exploring the interactome of PAR in breast cancer cells we identified NUDIX5 (also known as NUDT5) as a hormone-induced interactor. This member of the large NUDIX family of enzymes is known to hydrolyze ADPR to AMP and ribose-5-P and, in principle, it could use pyrophosphate (PPi) instead of H_2_O to convert ADPR to ATP and ribose-5-P. Therefore, we tested whether NUDIX5 was required for hormonal gene regulation. We found that depleting NUDIX5 by specific siRNAs resulted in a dramatic inhibition of hormonal gene regulation as well as of cell proliferation in response to either estrogens or progestins (Wright *et al.* 2016). Moreover, recombinant NUDIX5 can generate ATP when incubated with ADPR and PPi. Finally, using FRET or bioluminescence sensors for ATP visualization in living cells, we could detect a transient increase in nuclear ATP around 30 min after hormone exposure that returned to basal levels after 60 min (Wright *et al.* 2016) (Fig. 5A). Depleting NUDIX5 also resulted in an inhibition of hormone-induced chromatin remodeling and, therefore, we assumed that the nuclear ATP was required for the ATPases of the chromatin remodeling enzymes.

NUDIX5 is the only member of the NUDIX family that forms a homodimer (Zha *et al.* 2006). The NUDIX5 homodimer has two active clefts formed at the interface of the antiparallel oriented monomers. In the inactive state prior to hormone exposure, the monomers are tightly connected by ionic interaction between phosphorylated Threonine45 (T45) of one monomer and Lysine27 (K27) of the other (Wright *et al.* 2016). In this conformation, there is no space for PPi to enter the active cleft and the enzyme hydrolyzes ADPR (Fig. 5B, *left panel*). We wondered how NUDIX5 is activated upon hormone exposure and found that 1 min after adding hormone T45 is rapidly dephosphorylated (Wright *et al.* 2016) (Fig. 5B, *right panel*). Dephosphorylation of T45 weakens the interaction between the two monomers, allowing them to flip and form a hexamer that exhibits more open substrate clefts able to accept PPi (Wright *et al.* 2016). The phosphomimetic NUDIX5 mutant T45D cannot synthesize ATP and acts as dominant negative mutation on gene regulation (Wright *et al.* 2016). Thus, we have identified a novel nuclear pathway in which nicotinamide nucleotide adenylyltransferase 1 (NMNAT1) uses ATP and nicotinamide mononucleotide (NMN) to synthesize NAD^+^, that is used by PARP1 to attach PAR chains to itself and to chromatin proteins; PARG hydrolyses PAR to ADPR, which NUDIX5 can either hydrolyze to AMP or use ADPR along with PPi to synthesize ATP, depending on its phosphorylation state (Fig. 5C).

**Figure 6:**
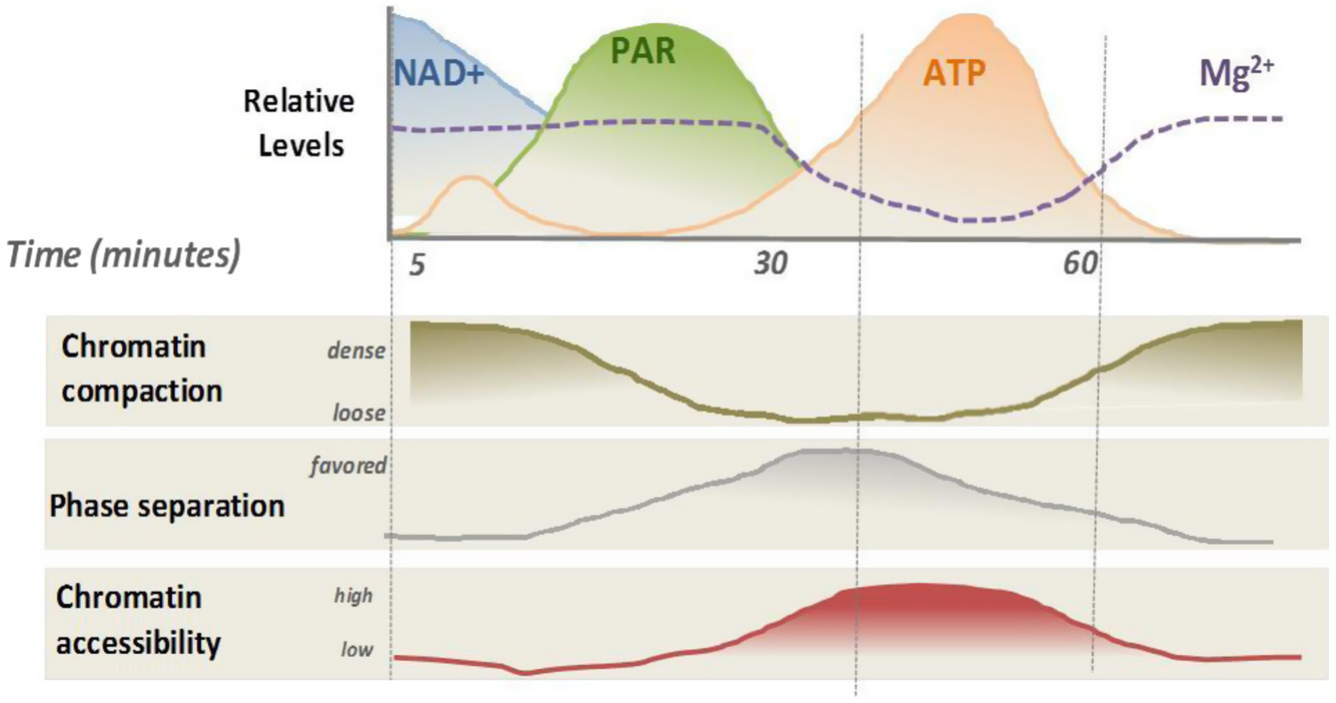
Time course of the nuclear levels of NAD^+^, PAR, ATP and Mg^2+^, correlated with chromatin accessibility changes. Hypothetical graph representing the changes in the nuclear levels of NAD^+^, PAR, ATP and Mg^2+^, as a function of time after hormone addition. Initially, when NAD^+^ is converted to PAR, possibly, part of the initial pool of nuclear ATP is used for chromatin remodeling, but the large increase in nuclear ATP after 30 min is not related to chromatin remodeling but most likely is used for chromatin decompaction as an hydrotrope and as a chelator of free Mg^2+^ ions. Once the ATP levels return to basal levels, the chromatin compacts again reducing the risk for DNA damage (Wright *et al.* 2016, 2019).

In interpreting these results, we were faced with a problem since chromatin remodeling is virtually finished 30 min after hormone exposure (Vicent *et al.* 2009), the time when we start to see accumulation of ATP. We reasoned that this was an excess of ATP not used by the chromatin remodeling ATPases and were wondering about its possible function. At that time a paper from Tony Hyman’s laboratory appeared in *Science* showing that ATP at millimolar concentrations could act as an hydrotrope that would facilitate high concentrations of macromolecules as required for macromolecular condensates and phase transitions (Patel *et al.* 2017). This appeared as a very plausible role for the large amount of nuclear ATP that we detected and that could serve to transiently facilitate chromatin transcription and RNA processing (Wright *et al.* 2019). Another possible function of mM ATP concentrations could be to bind free Mg^2+^ ions reducing their free concentration and thereby promoting chromatin decompaction (Maeshima *et al.* 2018). In fact, Mg^2+^ ions at mM concentrations neutralize the charge of the histone tails and have been used to precipitate chromatin in the past (Widom 1986). The energy expensive synthesis of NAD^+^ by MNAT1 in the nucleus using mitochondrial ATP and the conversion of NAD^+^ to PAR by PARP1 leads to the accumulation of large amount of chemical energy in ADPR. Part of this chemical energy is used for nuclear ATP synthesis by activated NUDIX5 to fulfill various consecutive nuclear functions, including ATP-dependent chromatin remodeling, likely also histone chaperones function, followed by chromatin decompaction as an hydrotrope and as a chelator of free Mg^2+^ ions (Wright *et al.* 2019) (Fig. 6). The availability of NUDIX5 specific inhibitors (Page *et al.* 2018) will help to clarify these processes in more sophisticated biological models, including 3D cell cultures and mouse intraductal xenografts (Sflomos *et al.* 2016). We have recently found that nuclear synthesis of ATP by NUDIX5 is essential for the formation of oncospheres by breast cancer cells grown in 3D cultures, due to the dependence of the cancer stem cells on nuclear ATP (Pickup *et al.* 2019). As NUDIX5 is overexpressed in breast tumors and correlates with poor prognosis (Wright *et al.* 2016), it represents a novel possible target for the pharmacological control of cancer growth. In addition, we know that nuclear ATP synthesis is needed for DNA damage repair (Wright *et al.* 2016). As cancer cells are addicted to DNA repair mechanisms, blocking NUDIX5 alone or in combination with PARP inhibitors could be another possible strategy for the pharmacological management of breast cancer.

## The next frontier: liquid-liquid phase transitions of macromolecular condensates

One aspect that is becoming increasingly relevant for the complete understanding of nuclear function is the formation of macromolecular condensates and liquid-liquid phase transitions (LLPT) (Peng and Weber 2019). Given that the cell nucleus has many different structures that are not separated by membranes, including the nucleolus, Cajal bodies, paraspeckles, transcription factories and the two phases of chromatin – euchromatin and heterochromatin – LLPT is a plausible mechanism for regulating the functions of these macromolecular condensates. In particular, chromatin as a long heavily charged polymer has intrinsic properties encoded mainly in the non-structured charged tails of linker and core histones. This will facilitate phase transitions in response to changes in ionic strength or to post-translational modifications of the histone tails, in particular acetylation of lysines (Gibson *et al.* 2019), but also methylation of lysines and arginines, citrullination of arginines, phosphorylation of serines and threonines and ADP-ribosylation of serines (Altmeyer *et al.* 2015; Larsen *et al.* 2018), or in response to binding of regulatory proteins (for example: transcription factors, readers of epigenetic marks or members of the basic transcriptional machinery including the CTD of RNA polymerase II) (Gibson *et al.* 2016). The discovery that the state of phosphorylation of the CTD can drive the transition from a transcriptional condensate to a splicing condensate (Guo *et al.* 2019) is relevant in view of the fact that hormonal regulation influences the state of the CTD not only in terms of its phosphorylation state, but also via PADI2-mediated citrullination of arginine 1810 that favors promoter release and elongation by RNA polymerase II (Sharma *et al.* 2019).

In relation to the action of PR, it is important to note that the two isoforms A and B may differ in the type of transcription factor and kinases with which they interact (Bellance *et al.* 2013; Kaya *et al.* 2015) and that the PRA isoform lacks one transactivation function (Tung *et al.* 2006). Methylation of K464 in the activation function 1 (AF1) activates PR (Chung *et al.* 2014) and the triple mutant K464, K481 and R492 to QQQ makes PR hyperactive in the absence of ligand (Woo *et al.* 2019), indicating relevant functional interactions of the AF1. This may be physiologically relevant since very recently it has been found that transgenic mice expressing only the PRB isoform develop ovarian neoplasms, while those expressing only PRA displayed a reduced frequency of tumor development (Wetendorf *et al.* 2019). We know that, as in the case of androgen receptor (De Mol *et al.* 2018), the N-terminal unstructured region of PR can form droplets *in vitro*. Therefore, it could be involved in cluster formation detected in cell nuclei in response to hormone that was different for the A and B isoforms of PR (Lim *et al.* 1999). We are now investigating how this physical behavior of PR may be combined with the modifications of the CTD of the RNA polymerase II and with changes in chromatin PARylation or other chromatin epigenetic marks, along with changes in nuclear ATP and Mg^2+^ ions, to facilitate the formation of macromolecular condensates that mediate hormonal gene regulation. In experiments with electron microscopy and immunogold, it was shown that PR binds preferentially to the condensed heterochromatin near the lamina and that, in response to hormone, there is disaggregation of the condensed chromatin to smaller fragments and the immunogold particles accumulate in the border between this dispersed chromatin and the nucleoplasm (Perrot-Applanat *et al.* 1986). The recent advances in high resolution microscopy and single molecule tracking will be key for exploring the dynamics of nuclear phase transitions in living cells. In particular, the transitions between the active and the inactive states of chromatin can be studied using soft X-ray tomography (Le Gros *et al.* 2016; Strom *et al.* 2017) and may be correlated with visualization of specific chromatin regions via oligo-painting and oligoSTORM (Beliveau *et al.* 2015; Beliveau *et al.* 2017) and localization of PR tagged with an appropriate fluorophore. Combined with single-cell advanced studies (Jin *et al.* 2015), approaches like combinatorial cellular indexing (Cusanovich *et al.* 2015), RNA seqFISH (Eng *et al.* 2019), single cell Hi-C (Nagano *et al.* 2017; Ramani *et al.* 2017; Stevens *et al.* 2017), single cell MNase-seq (Lai *et al.* 2018), itChIP-seq (Ai *et al.* 2019), Dip-C (Tan *et al.* 2019) or CHIA-DROP (Zheng *et al.* 2019) will permit insight into the mechanism that uses dynamic variability in structure (Finn and Misteli 2019) and the stochastic nature of gene transcription (Raser and O’Shea 2004) to permit adaptation of the cell to changes in the environment, such as the exposure to variable levels of steroid hormones.

### Acknowledgements

First, the authors thank all the members of the Chromatin and Gene Expression Group, who had performed most of the experiments commented in this review and have made suggestions for the text. The authors also thank their collaborators, most of them cited as authors of the referenced papers. The authors thank the CRG for the continuous support of the group and for the availability of essential core facilities. The experimental work mentioned was supported by the core funding of the CRG, the European Research Council (Project ‘4D Genome’ 609989), the Ministerio de Economía y Competitividad (Project G62426937) and the Generalitat de Catalunya (Project AGAUR SGR 575). We acknowledge support of the Spanish Ministry of Economy, Industry and Competitiveness (MEIC) to the EMBL partnership, the Centro de Excelencia Severo Ochoa as well as CERCA Programme / Generalitat de Catalunya.

## Declaration of interest

The authors declare that there is no conflict of interest that could be perceived as prejudicing the impartiality of the research reported.
